# Polymorphisms of the Toll-Like Receptor-3 Gene in Autoimmune Adrenal Failure and Type 1 Diabetes in Polish Patients

**DOI:** 10.1007/s00005-015-0360-z

**Published:** 2015-08-30

**Authors:** Marta Fichna, Magdalena Żurawek, Piotr Fichna, Danuta Januszkiewicz-Lewandowska, Marek Ruchała, Jerzy Nowak

**Affiliations:** Institute of Human Genetics, Polish Academy of Sciences, Strzeszynska 32, 60-479 Poznan, Poland; Department of Endocrinology, Metabolism and Internal Medicine, Poznan University of Medical Sciences, Poznan, Poland; Department of Clinical Immunology, Poznan University of Medical Sciences, Poznan, Poland; Department of Paediatric Diabetes and Obesity, Poznan University of Medical Sciences, Poznan, Poland

**Keywords:** Addison’s disease, Polymorphism, TLR3, Type 1 diabetes

## Abstract

Infectious agents are plausible environmental triggers for autoimmunity in genetically susceptible individuals. Polymorphic variants of genes implicated in innate immunity may affect immune responses and hence promote auto-aggressive reactions. Genes such as Toll-like receptor-3 (*TLR3*), which participate in recognizing conserved foreign molecules and mounting the first line of defence against viral infections, are promising functional candidates in autoimmune conditions. We investigated the association of the *TLR3* variants, rs13126816 and rs3775291, with the autoimmune endocrine disorders, Addison’s disease (AD) and type 1 diabetes (T1D) in the Polish population. The study comprised 168 AD patients, 524 individuals with T1D and 592 healthy controls. Genotyping was performed by real-time PCR. Distribution of the *TLR3* genotypes and alleles did not reveal significant differences between patients and controls (*p* > 0.05). No effect on age at disease onset was found in affected cohorts. This analysis does not support an association between *TLR3* variants and the risk for autoimmune destruction of the adrenal cortex and beta cells. However, innate immunity merits further studies in autoimmune endocrine conditions.

## Introduction

Type 1 diabetes (T1D), which affects the insulin-producing beta cells of the pancreas, and Addison’s disease (AD), which results from the destruction of the adrenal cortex, are life-threatening autoimmune conditions, leading to the absolute dependence on exogenous hormones supply. Unfortunately, in the vast majority of cases, the origin of the auto-aggressive phenomena remains obscure. It has been proposed that the autoimmune reactions are triggered in genetically predisposed subjects on encounter of some environmental factors, which may promote aberrant immune reactivity (Michels and Eisenbarth 2010). There is a mounting body of evidence in support of the role of the viral infections, notably by enteroviruses, in the development of T1D. Enteroviruses present tropism for pancreatic islets and enterovirus antibodies and RNA are found in newly diagnosed T1D patients more frequently than in their healthy peers (Tauriainen et al. 2011; Yeung et al. 2011). The environmental influence in aetiology of AD is even less recognized. Some authors have reported its relationship with herpesvirus infections and recent experimental data corroborate that virus-induced type I and III interferons (IFNs) might initiate or enhance an ongoing autoimmune reaction (Hellesen et al. 2014; Schmitt et al. 1996).

Mechanisms of the non-specific immunity start to draw increasing attention in the context of autoimmune disorders (Meyers et al. 2010). According to the association studies, innate immunity genes—*CIITA*, *NLRP1* and *IFIH1*—may be implicated in beta cell and adrenal cortex autoimmunity (Magitta et al. 2009; Skinningsrud et al. 2008; Smyth et al. 2006). They belong to the pattern recognition receptors (PRRs) that recognize the conserved microbial structures and induce the first line of defence (Thompson et al. 2011). PRRs also comprise the family of the Toll-like receptors (TLRs), which were first identified for their role in embryonic development in *Drosophila*, but subsequently appeared important elements of innate immunity in adult flies, with their homologs found across the evolutionary spectrum up to humans (Kawai and Akira 2010). Considering data from other PRRs, it seemed plausible that TLRs, especially those sensing viral nucleic acids, might equally harbour susceptibility loci for autoimmune conditions. Indeed, variations within the *TLR3* locus (4q35) are associated with predisposition to viral infections and to systemic autoimmune diseases (Laska et al. 2014; Qian et al. 2013; Svensson et al. 2012). TLR3 is an endosomal receptor, which recognizes double-stranded RNA (dsRNA), an intermediate during the replication of most viruses, and triggers immune responses by stimulating the synthesis of type I IFNs and inflammatory cytokines (Alexopoulou et al. 2001). Not only is it expressed in a variety of immune cells, but also found in human and rodent pancreatic beta cells and adrenal cortex (Alexopoulou et al. 2001; Hultcrantz et al. 2007; Kanczkowski et al. 2009). A study in a small South African cohort suggested an association between T1D and polymorphisms at the *TLR3* gene (Pirie et al. 2005). This observation was further substantiated in a recent analysis of Brazilian population although T1D-associated single nucleotide polymorphisms (SNPs) did not overlap with the former African findings (Assmann et al. 2014). On the contrary, data from the Han Chinese do not support the association between *TLR3* and T1D in Asians; however, only two gene variants were explored (Sun et al. 2014). Polymorphisms of *TLR3* have not been analysed in AD and in European T1D cohorts to date. Our study was, therefore, designed to investigate the association of selected *TLR3* SNPs with these autoimmune endocrine disorders among Polish subjects.

## Materials and Methods

Two variants of the *TLR3* gene were genotyped in a cohort of 168 AD patients, 524 individuals with T1D and in 592 healthy controls issued from Caucasian Polish population. The diagnosis of T1D was based upon WHO criteria with an absolute dependence on exogenous insulin. Clinical diagnosis of adrenal failure was confirmed by either low basal serum cortisol with a high adrenocorticotropin level, or subnormal response to the short Synacthen test (Husebye et al. 2014). Autoimmune aetiology was corroborated by positive serum autoantibodies to insulin, glutamic acid decarboxylase and/or islet antigen IA2 in T1D, and autoantibodies to 21-hydroxylase in AD. Mean age at AD onset was 32.6 (±10.9) years and mean age at T1D onset was 8.5 (±4.4) years. Patients were enrolled at the endocrine clinics of the Poznan University of Medical Sciences. Control subjects were recruited among healthy blood donors with negative history and no signs of autoimmune disorders. The local ethical board approved the study and informed consent was obtained from its participants.

Genomic DNA was extracted from the peripheral blood using Gentra Puregene Blood Kit (Qiagen, Hilden, Germany). Genotyping of rs13126816 and rs3775291 was performed by real-time PCR using commercial Taqman assays (C_32209947 and C_1731425_10). Allelic discrimination analyses were carried out using 7900 HT Fast Real-Time PCR System (Applied Biosystems, Foster City, CA, USA) and Sds2.3 software. Genotypes were confirmed by direct DNA sequencing of both strands by BigDye Terminator Cycle Sequencing Ready Reaction Kit on ABI Prism 3730 Genetic Analyzer (Foster City, CA, USA) and controls were used in all genotyping reactions. To ensure fidelity, 8 % of samples were re-genotyped blind.

The study was designed as a replication of Brazilian findings in T1D, which revealed odds ratios (ORs) of 2.1 and 2.3 for rs13126816 and rs3775291, respectively. The power estimation, performed with PS Power and Sample Size calculator v.2.1.30 (Vanderbilt University, TN, USA) assuming an allelic OR of 1.5 and given the minor allele frequencies as observed in the control group, showed >99 % power to detect effects of both studied SNPs in our T1D cohort and 87.8 and 89.9 % power to detect the respective effects of rs13126816 and rs3775291 in AD (*α* = 0.05).

Genotypes were checked for Hardy–Weinberg equilibrium (threshold *p* > 0.05) using an online calculator available at the Helmholtz Center Munich website (http://ihg.gsf.de/cgi-bin/hw/hwa1.pl). *χ*^2^ test was used for association analysis on 2 × 2 and 2 × 3 contingency tables. Linkage disequilibrium (LD) measures, Lewontin’s *D*′ and *r*^2^ coefficient, were calculated using Haploview v.4.1 (Broad Institute of Harvard and MIT, Cambridge, MA, USA). Haplotype frequencies were estimated based on maximum likelihood method and compared between patients and controls (*χ*^2^ test). Genotype-stratified normally distributed data on age at disease onset were compared using one-way ANOVA and those with non-normal distribution were analysed by Kruskal–Wallis test. Statistical calculations were performed using SPSS 18.0 software (SPSS Inc., Chicago, IL, USA).

## Results

Both analysed polymorphisms were in Hardy–Weinberg equilibrium in all studied cohorts (*p* > 0.150). The frequencies of alleles and genotypes of the two *TLR3* variants did not present significant differences between patients and controls (Table 1).

**Table 1:** Distribution of the *TLR3* polymorphisms rs13126816 and rs3775291 in patients with Addison’s disease (AD), type 1 diabetes (T1D) and healthy controls (CON)

Polymorphism	Genotypes	Alleles	AD *n* (%)	T1D *n* (%)	CON *n* (%)
rs13126816	GG		104 (61.9)	316 (60.3)	345 (58.3)
	AG		57 (33.9)	181 (34.5)	205 (34.6)
	AA		7 (4.2)	27 (5.2)	42 (7.1)
			*p* = 0.359	*p* = 0.389	
		G	265 (78.9)	813 (77.6)	895 (75.6)
		A	71 (21.1)	235 (22.4)	289 (24.4)
	*p* value		0.212	0.269	
	OR (95 % CI)		0.829 (0.619–1.113)	0.895 (0.735–1.090)	
rs3775291	GG		74 (44.1)	253 (48.3)	292 (49.3)
	AG		76 (45.2)	232 (44.3)	236 (39.9)
	AA		18 (10.7)	39 (7.4)	64 (10.8)
			*p* = 0.432	*p* = 0.092	
		G	224 (66.7)	738 (70.4)	820 (69.3)
		A	112 (33.3)	310 (29.6)	364 (30.7)
	*p* value		0.366	0.550	
	OR (95 % CI)		1.126 (0.870–1.458)	0.946 (0.789–1.134)	

*p* values represent comparison between patients and controls

*OR* odds ratio, *CI* confidence interval

Linkage disequilibrium evaluation revealed moderate LD between studied SNPs, with the *D*′ values ranging between 0.69 and 0.76 and the *r*^2^ values between 0.24 and 0.43 in investigated cohorts. The analysis of the inferred two-allele haplotypes demonstrated increased frequency of the rs13126816/G–rs3775291/A haplotype in AD subjects (*p* = 0.002) and a similar borderline trend among T1D patients (*p* = 0.055) compared to controls (Table 2).

**Table 2:** Distribution of the *TLR3* bi-allelic haplotypes (rs13126816–rs3775291) in patients with Addison’s disease (AD), type 1 diabetes (T1D) and healthy controls (CON)

	AD (2n = 336)	*p* value	T1D (2n = 1048)	*p* value	CON (2n = 1184)
GG	208 (61.9)	Reference	686 (65.4)	Reference	773 (65.3)
AA	58 (17.3)	0.425	178 (17.0)	0.067	246 (20.8)
GA	55 (16.4)	0.002	136 (13.0)	0.055	118 (10.0)
AG	15 (4.4)	0.577	48 (4.6)	0.507	47 (3.9)

*p* values represent comparison between patients and controls (GG—reference haplotype)

No influence of the polymorphic *TLR3* variants on the age at disease diagnosis was detected in either AD or T1D cohort (*p* = 0.870 and *p* = 0.731 for comparisons between three rs13126816 genotypes and *p* = 0.636 and *p* = 0.074 for comparisons between three rs3775291 genotypes, respectively).

## Discussion

Numerous data from the animal models treated with synthetic dsRNA analogue, polyinosinic–polycytidylic acid [poly(I:C)], support the hypothesis that disturbed TLR3 function may contribute to the development of T1D in susceptible individuals (Alkanani et al. 2014; Devendra et al. 2005; Moriyama et al. 2002; Sobel et al. 1992). Recent findings from mice, which present widespread apoptosis of the pancreatic cells in response to a rotavirus infection, indicate that the detrimental viral effect on the pancreas is initially mediated by the dsRNA–TLR3 interaction (Honeyman et al. 2014). Furthermore, it has been demonstrated that virus-infected or IFN-stimulated human islets displayed increased TLR3 expression (Hultcrantz et al. 2007; Sarmiento et al. 2013). With respect to adrenal autoimmunity, TLR3-expressing adrenocortical carcinoma cells stimulated with poly(I:C) along with IFN-γ or tumor necrosis factor *α* display significant rise in production of CXCL10, a chemokine involved in autoimmune adrenal failure (Bratland et al. 2013). These data suggest that TLR3 may be an important player in autoimmune endocrinopathy and its altered function could contribute to disease development. Unfortunately, the current study does not support an association between *TLR3* polymorphisms and autoimmune endocrine conditions in Polish patients. Despite slightly larger T1D and control cohorts, we were unable to replicate the Brazilian findings and we could not demonstrate an association of rs13126816 or rs3775291 with AD either. The power of the current study exceeded recommended 80 % when calculated for an OR of 1.5; however, it could still be insufficient to detect a smaller effect (Hong and Park 2012).

The initial analysis of the *TLR3* region in T1D comprised several SNPs evaluated in a cohort of 79 South African patients. Significant associations were found for three variants: rs5743313, rs5743315 and 2690 A/G but none of them survived multiple-comparisons correction (Pirie et al. 2005). In the subsequent Brazilian study, the choice of investigated polymorphisms was based on linkage disequilibrium pattern across *TLR3* region and previously known associations with various immune conditions (Assmann et al. 2014). Two variants (rs13126816 and rs3775291) appeared significantly associated with T1D (Assmann et al. 2014). Rs13126816 in the long intron 1 had been previously correlated with the expression of TLR3, clearance of hepatitis C virus, rubella virus-specific cytokine responses, and susceptibility to herpesvirus-2 infection (Ovsyannikova et al. 2010; Qian et al. 2013; Svensson et al. 2012). The non-synonymous rs3775291 (Leu412Phe) located in exon 4 confers reduced binding capacity to dsRNA and is associated with susceptibility to viral infections and systemic autoimmunity (Barkhash et al. 2013; Laska et al. 2014; Sironi et al. 2012; Svensson et al. 2012). On the contrary, our study failed to detect any association of the rs13126816 and rs3775291 with the organ-specific autoimmune conditions. However, haplotype analysis suggests that combined allele effect might play a role. In line, the frequency of the disease in Brazilians appeared to rise with the overall number of the *TLR3* risk alleles. Nonetheless, the rs3775291 T1D risk allele from the Brazilian study was opposite to the one, which could plausibly contribute to AD susceptibility in our cohort and which is connected with systemic autoimmune conditions and resistance to viral infections (Laska et al. 2014; McCartney et al. 2011; Svensson et al. 2012). Discrepant allele effects are not uncommon in case–control studies and usually explained on the basis of the population differences (Fichna et al. 2015; Owen et al. 2007). Considering multi-ethnic Brazilian society and the fact that study participants were only self-defined as white (Assmann et al. 2014), population stratification might be implicated (Lander and Schork 1994). Moreover, Brazilian control group displayed distinct allele frequencies compared to healthy subjects in other Caucasian populations, including our cohort (rs13126816/A found in 38 % Brazilians vs. 26.9 % in CEU HapMap sample, and rs3775291/A in 37 % Brazilian controls vs. 25.4–33.7 % in other Caucasians) (Assmann et al. 2014; Kindberg et al. 2011; Laska et al. 2014; Sironi et al. 2012). Finally, there might be another causative *TLR3* variant implicated in autoimmune endocrine conditions, and differences in LD pattern across various populations could contribute to discrepant results for rs13126816 and rs3775291. However, genuine lack of *TLR3* association with T1D is further underpinned by the results of the genome-wide scans in the large Caucasian cohorts, with the closest association localized to the interleukin-2 gene at 4q27 (Barrett et al. 2009; Smyth et al. 2006; Todd et al. 2007; Wellcome Trust Case Control Consortium 2007).

In conclusion, *TLR3* polymorphisms are not likely to be associated with the risk for T1D and AD in Polish population. However, considering increasing evidence of interference between innate immunity and self-aggressive phenomena, it may still be of interest to investigate a larger set of innate immunity genes in the autoimmune endocrine conditions in future.

## References

[CR1] Alexopoulou L, Holt AC, Medzhitov R (2001). Recognition of double-stranded RNA and activation of NF-kappaB by Toll-like receptor 3. Nature.

[CR2] Alkanani AK, Hara N, Lien E (2014). Induction of diabetes in the RIP-B7.1 mouse model is critically dependent on TLR3 and MyD88 pathways and is associated with alterations in the intestinal microbiome. Diabetes.

[CR3] Assmann TS, de Brondani L, Bauer AC (2014). Polymorphisms in the TLR3 gene are associated with risk for type 1 diabetes mellitus. Eur J Endocrinol.

[CR4] Barkhash AV, Voevoda MI, Romaschenko AG (2013). Association of single nucleotide polymorphism rs3775291 in the coding region of the TLR3 gene with predisposition to tick-borne encephalitis in a Russian population. Antiviral Res.

[CR5] Barrett JC, Clayton DG, Concannon P (2009). Genome-wide association study and meta-analysis find that over 40 loci affect risk of type 1 diabetes. Nat Genet.

[CR6] Bratland E, Hellesen A, Husebye ES (2013). Induction of CXCL10 chemokine in adrenocortical cells by stimulation through toll-like receptor 3. Mol Cell Endocrinol.

[CR7] Devendra D, Jasinski J, Melanitou E (2005). Interferon-alpha as a mediator of polyinosinic:polycytidylic acid-induced type 1 diabetes. Diabetes.

[CR8] Fichna M, Żurawek M, Bratland E (2015). Interleukin-2 and subunit alpha of its soluble receptor in autoimmune Addison’s disease—an association study and expression analysis. Autoimmunity.

[CR9] Hellesen A, Edvardsen K, Breivik L (2014). The effect of types I and III interferons on adrenocortical cells and its possible implications for autoimmune Addison’s disease. Clin Exp Immunol.

[CR10] Honeyman MC, Laine D, Zhan Y (2014). Rotavirus infection induces transient pancreatic involution and hyperglycemia in weaning mice. PLoS ONE.

[CR11] Hong EP, Park JW (2012). Sample size and statistical power calculation in genetic association studies. Genomics Inform.

[CR12] Hultcrantz M, Huhn MH, Wolf M (2007). Interferons induce an antiviral state in human pancreatic islet cells. Virology.

[CR13] Husebye ES, Allolio B, Arlt W (2014). Consensus statement on the diagnosis, treatment and follow-up of patients with primary adrenal insufficiency. J Intern Med.

[CR14] Kanczkowski W, Zacharowski K, Wirth MP (2009). Differential expression and action of Toll-like receptors in human adrenocortical cells. Mol Cell Endocrinol.

[CR15] Kawai T, Akira S (2010). The role of pattern-recognition receptors in innate immunity: update on Toll-like receptors. Nat Immunol.

[CR16] Kindberg E, Vene S, Mickiene A (2011). A functional Toll-like receptor 3 gene (TLR3) may be a risk factor for tick-borne encephalitis virus (TBEV) infection. J Infect Dis.

[CR17] Lander ES, Schork NJ (1994). Genetic dissection of complex traits. Science.

[CR18] Laska MJ, Hansen B, Troldborg A (2014). A non-synonymous single-nucleotide polymorphism in the gene encoding Toll-like Receptor 3 (TLR3) is associated with sero-negative Rheumatoid Arthritis (RA) in a Danish population. BMC Res Notes.

[CR19] Magitta NF, Boe Wolff AS, Johansson S (2009). A coding polymorphism in NALP1 confers risk for autoimmune Addison’s disease and type 1 diabetes. Genes Immun.

[CR20] McCartney SA, Vermi W, Lonardi S (2011). RNA sensor-induced type I IFN prevents diabetes caused by a β cell-tropic virus in mice. J Clin Invest.

[CR21] Meyers AJ, Shah RR, Gottlieb PA (2010). Altered Toll-like receptor signaling pathways in human type 1 diabetes. J Mol Med.

[CR22] Michels AW, Eisenbarth GS (2010). Immunologic endocrine disorders. J Allergy Clin Immunol.

[CR23] Moriyama H, Wen L, Abiru N (2002). Induction and acceleration of insulitis/diabetes in mice with a viral mimic (polyinosinic–polycytidylic acid) and an insulin self-peptide. Proc Natl Acad Sci USA.

[CR24] Ovsyannikova IG, Dhiman N, Haralambieva IH (2010). Rubella vaccine-induced cellular immunity: evidence of associations with polymorphisms in the Toll-like, vitamin A and D receptors, and innate immune response genes. Hum Genet.

[CR25] Owen CJ, Kelly H, Eden JA (2007). Analysis of the Fc receptor-like-3 (FCRL3) locus in Caucasians with autoimmune disorders suggests a complex pattern of disease association. J Clin Endocrinol Metab.

[CR26] Pirie FJ, Pegoraro R, Motala AA (2005). Toll-like receptor 3 gene polymorphisms in South African Blacks with type 1 diabetes. Tissue Antigens.

[CR27] Qian F, Bolen CR, Jing C (2013). Impaired toll-like receptor 3-mediated immune responses from macrophages of patients chronically infected with hepatitis C virus. Clin Vaccine Immunol.

[CR28] Sarmiento L, Frisk G, Anagandula M (2013). Expression of innate immunity genes and damage of primary human pancreatic islets by epidemic strains of Echovirus: implication for post-virus islet autoimmunity. PLoS ONE.

[CR29] Schmitt K, Deutsch J, Tulzer G (1996). Autoimmune hepatitis and adrenal insufficiency in an infant with human herpesvirus-6 infection. Lancet.

[CR30] Sironi M, Biasin M, Cagliani R (2012). A common polymorphism in TLR3 confers natural resistance to HIV-1 infection. J Immunol.

[CR31] Skinningsrud B, Husebye ES, Pearce SH (2008). Polymorphisms in CLEC16A and CIITA at 16p13 are associated with primary adrenal insufficiency. J Clin Endocrinol Metab.

[CR32] Smyth DJ, Cooper JD, Bailey R (2006). A genome-wide association study of nonsynonymous SNPs identifies a type 1 diabetes locus in the interferon-induced helicase (IFIH1) region. Nat Genet.

[CR33] Sobel DO, Newsome J, Ewel CH (1992). Poly I: C induces development of diabetes mellitus in BB rat. Diabetes.

[CR34] Sun C, Zhi D, Shen S (2014). SNPs in the exons of Toll-like receptors are associated with susceptibility to type 1 diabetes in Chinese population. Hum Immunol.

[CR35] Svensson A, Tunback P, Nordstrom I (2012). Polymorphisms in Toll-like receptor 3 confer natural resistance to human herpes simplex virus type 2 infection. J Gen Virol.

[CR36] Tauriainen S, Oikarinen S, Oikarinen M (2011). Enteroviruses in the pathogenesis of type 1 diabetes. Semin Immunopathol.

[CR37] Thompson MR, Kaminski JJ, Kurt-Jones EA (2011). Pattern recognition receptors and the innate immune response to viral infection. Viruses.

[CR38] Todd JA, Walker NM, Cooper JD (2007). Robust associations of four new chromosome regions from genome-wide analyses of type 1 diabetes. Nat Genet.

[CR39] Wellcome Trust Case Control Consortium (2007). Genome-wide association study of 14,000 cases of seven common diseases and 3,000 shared controls. Nature.

[CR40] Yeung WC, Rawlinson WD, Craig ME (2011). Enterovirus infection and type 1 diabetes mellitus: systematic review and meta-analysis of observational molecular studies. BMJ.

